# Digital Healthcare Approaches for Fall Detection and Prediction in Older Adults: A Systematic Review of Evidence from Hospital and Long-Term Care Settings

**DOI:** 10.3390/medicina61111926

**Published:** 2025-10-27

**Authors:** Aijin Lee, Haneul Lee, Seon-Heui Lee

**Affiliations:** 1 Graduate School, College of Nursing, Gachon University, 191 Hambakmoe-ro, Yeonsu-gu, Incheon 21936, Republic of Korea; aijin9409@naver.com; 2 Department of Physical Therapy, College of Medical Science, Gachon University, 191 Hambakmoe-ro, Yeonsu-gu, Incheon 21936, Republic of Korea; 3 Research Institute of AI and Nursing Science, College of Nursing, Gachon University, 191 Hambakmoe-ro, Yeonsu-gu, Incheon 21936, Republic of Korea

**Keywords:** older adults, digital health, fall prevention, fall detection and prediction, systematic review

## Abstract

*Background and Objectives*: Falls are a leading cause of morbidity and mortality among older adults in hospitals and long-term care facilities (LTCFs). Digital healthcare approaches are increasingly being applied to fall detection and prevention; however, their effectiveness remains uncertain. This review evaluated the effectiveness, usability, and clinical applicability of detection- and prediction-based systems in institutional settings. *Materials and Methods*: We systematically searched major international and Korean databases—PubMed, Embase, Ovid-MEDLINE, CINAHL, the Cochrane Library, IEEE, KMbase, KISS, KoreaMed, and RISS—for studies published up to December 2024. The eligible studies included randomized controlled trials, quasi-experimental, and observational studies involving older adults in hospitals or LTCFs. Two reviewers independently screened the studies, extracted data, and assessed their quality using standardized tools. *Results*: Thirty-three studies comprising 20 fall detection systems and 13 fall prediction models were included. Detection systems using inertial, pressure, radar, or multimodal sensors have improved monitoring and achieved high usability (>80% acceptance); however, they did not consistently reduce fall incidence or the occurrence of injurious falls. For instance, one trial reported a nonsignificant reduction in injurious falls (aRR 0.56, 95% CI 0.17–1.79), whereas another trial observed a nonsignificant increase (aIRR 1.60, 95% CI 0.83–3.08). Frequent false alarms contribute to alarm fatigue. The prediction models showed moderate-to-strong discrimination. Gradient boosting and neural networks performed best for continuous gait features, while regression and boosting approaches were effective for categorical EHR data. Most models lacked external validation and were not linked to clinical care pathways. *Conclusions*: Digital approaches show potential for fall prevention in hospitals and LTCFs; however, current evidence remains inconsistent and limited. Detection systems improve surveillance but offer limited preventive effects, whereas prediction models demonstrate technical promise without establishing clinical benefits. Future research should refine the technology, validate models externally, and integrate them into patient-centered workflows.

## 1. Introduction

Falls are among the most common and serious patient safety worldwide, particularly among older adults. According to the World Health Organization, approximately 37 million falls requiring medical attention occur annually, making falls the second leading cause of unintentional injury deaths worldwide [1]. In hospital settings, the incidence of inpatient falls has been reported to be 3–5 per 1000 bed-days, with up to 1 million cases occurring annually [2]. These figures highlight the substantial risk posed by falls in acute and institutional care environments and underscore the urgent need for effective prevention strategies.

Beyond immediate injuries such as fractures, head trauma, and increased mortality, falls often result in long-term functional decline, loss of independence, and an increased risk of institutionalization [3,4]. Psychological consequences, including a fear of falling and reduced confidence in one’s mobility, can exacerbate social isolation and depression [5]. Falls also impose a substantial economic burden on healthcare systems, with direct costs from acute care, surgery, and rehabilitation, as well as indirect costs from extended caregiving and productivity loss [6]. Collectively, these outcomes illustrate why fall prevention is increasingly recognized as a global public health priority.

Older adults admitted to hospitals or residing in long-term care facilities (LTCFs) are at particularly high risk. Residents in these settings are typically older, have multiple chronic conditions, and are often prescribed several medications simultaneously, which increases their susceptibility to dizziness and balance impairments. Limited mobility, cognitive decline, and frailty increase the risk of falls. Importantly, falls in these environments not only lead to severe medical complications but also increase caregiver burden, staff workload, and institutional liability [7]. Therefore, preventing falls in institutionalized older populations is critical for maintaining patient safety and the quality of care.

Traditional fall risk assessments, including the Morse Fall Scale, Schmid Fall Risk Assessment, and Hendrich II Fall Risk Model, are widely used in clinical practice [8]. However, their predictive validity has been inconsistent, with some studies reporting a limited ability to differentiate fall incidence between high- and low-risk groups [9,10]. Moreover, a significant proportion of falls occur in patients initially classified as low-risk, highlighting the limitations of conventional tools [11]. These findings suggest that standard risk assessments alone are insufficient for effective fall prevention among institutionalized older adults.

In response, digital healthcare technologies have emerged as promising solutions. Advances in information and communication technology, wearable sensors, motion detection systems, and pressure-sensitive floor mats have enabled continuous monitoring and real-time detection of fall risk [12,13]. Machine learning algorithms and electronic medical record-based prediction models have also been developed to support the early identification of high-risk patients and individualized interventions [14]. The COVID-19 pandemic has accelerated the adoption of digital healthcare, expanding its application to remote monitoring and telehealth services [15]. Together, these innovations offer opportunities to address the shortcomings of conventional approaches by providing proactive, real-time, patient-centered fall prevention strategies.

Despite these advances, the evidence for digital fall prevention approaches remains fragmented. Some trials have demonstrated a reduced incidence of falls with sensor-based monitoring or ICT-supported programs, whereas others have reported inconsistent results or methodological weaknesses [16,17]. Furthermore, previous systematic reviews have often targeted specific populations, such as patients with dementia or cognitive impairment [18]; focused on a single type of intervention, such as bed/chair alarms or sensor systems in hospitalized patients [19]; or addressed only prediction-oriented approaches [16], thereby limiting their relevance to institutionalized older adults and recent technological advances. To address this gap, this systematic review aimed to evaluate the characteristics and effectiveness of digital healthcare approaches, including detection systems and prediction models, for fall prevention among older adults in hospital and LTCF settings.

## 2. Methods

### 2.1. Protocol and Registration

This systematic review was conducted in accordance with the Preferred Reporting Items for Systematic Reviews and Meta-Analyses (PRISMA) guidelines [20]. The protocol was registered with the International Prospective Register of Systematic Reviews (PROSPERO; CRD42024576340, registered on 16 August 2024). This study was exempt from review by the Institutional Review Board of Gachon University (No. 1044396-202408-HR-134-01).

### 2.2. Search Strategy

We searched for studies published up to December 2024 that examined digital approaches to fall detection and prediction in older adults in hospital and LTCF settings. The following electronic databases were used: Korean Medical Database (KMbase), Korean Studies Information Service System (KISS), KoreaMed, Research Information Sharing Service (RISS), Cumulative Index to Nursing and Allied Health Literature (CINAHL), Cochrane Library, Institute of Electrical and Electronics Engineers Xplore (IEEE), Ovid-MEDLINE, Ovid-Embase, and PubMed. The reference lists of the included studies were manually screened to identify additional relevant articles. Two independent researchers identified and selected keywords based on database-specific terminology, such as Medical Subject Headings (MeSH), Emtree terms, and text words. No language restrictions were applied in the database search; however, only studies in English and Korean were retained.

Keywords, along with database-specific controlled vocabulary, were combined using Boolean operators (AND and OR) to construct the final queries for each database. The search strategy was structured around the PICO framework, focusing on the population (older adults in hospitals or LTCF settings) and interventions (digital approaches to fall prevention). Comparison and Outcome terms were deliberately excluded from the core search string to maximize sensitivity and minimize the risk of missing relevant studies. Instead, these elements were applied during the study selection and data extraction. A detailed search strategy is provided in Supplementary S1.

### 2.3. Study Selection

Two researchers independently established the study selection criteria. The eligible studies met the predefined inclusion and exclusion criteria (Table 1). We included studies that (1) applied digital approaches for fall prevention, (2) involved older adults (≥60 years) admitted to or residing in hospitals or LTCFs, and (3) reported appropriate outcomes related to the effectiveness of digital healthcare approaches. The exclusion criteria were as follows: (1) studies not involving human participants; (2) duplicate publications; (3) conference abstracts, posters, protocols, and systematic reviews; (4) studies without available full text or relevant outcomes; and (5) studies not published in English or Korean.

Two researchers independently screened the titles and abstracts and then reviewed the full texts to confirm their eligibility. Disagreements were resolved through discussions with a third researcher. The screening process was conducted using Microsoft Excel spreadsheets to organize records and ensure an independent review.

### 2.4. Quality Assessment

The methodological quality of the included studies was assessed according to the study design using the Risk of Bias 2 (RoB 2.0) tool [21], the Mixed Methods Appraisal Tool (MMAT) [22], the Quality Assessment of Diagnostic Accuracy Studies-2 (QUADAS-2) tool [23], and the Prediction model Risk of Bias Assessment Tool (PROBAST) [24].

For randomized RCTs, the RoB 2.0 tool was used to evaluate five domains: bias arising from the randomization process, deviations from intended approaches, missing outcome data, measurement of outcomes, and selection of reported results. Each domain was rated as “low risk,” “some concerns,” or “high risk,” and the overall bias was derived from domain-level judgments [21]. For studies that employed mixed-method designs, the MMAT was used to assess methodological rigor across qualitative, quantitative, and integrated components, allowing for the evaluation of the appropriateness of data collection, analysis, and coherence of interpretations [22]. For diagnostic accuracy studies, the QUADAS-2 tool was used to evaluate both the risk of bias and applicability across four domains: patient selection, index test, reference standard, and flow and timing. Each domain was rated as “low,” “high,” or “unclear” risk of bias, and applicability concerns were assessed for the first three domains [23]. For the prediction model studies, the PROBAST tool was used to assess four domains: participants, predictors, outcomes, and analysis. Each domain was rated as “low,” “high,” or “unclear” for risk of bias, and issues such as model calibration and overfitting were considered when applicable [24].

In cases where diagnostic studies employed a randomized, quasi-experimental, or observational design, the appropriate combination of RoB 2.0 and QUADAS-2 tools was applied.

No studies were excluded based on quality ratings; instead, studies of lower quality were retained and their methodological limitations were considered in the synthesis and interpretation of findings [25].

## 3. Results

### 3.1. Search Results

**Figure 1:**
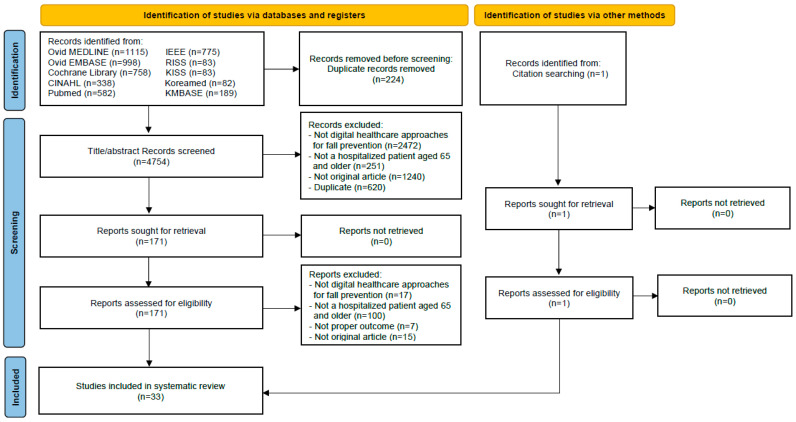
PRISMA flow chart of selected studies.

A total of 4978 records were identified. After removing 224 duplicates, 4754 titles and abstracts were screened, of which 171 were selected for full-text review. Following the full-text assessment, 138 articles were excluded, and one additional study was identified through a citation search. Finally, 33 studies were included in this systematic review. At the full-text screening stage, studies were excluded for the following primary reasons: (1) they did not involve digital healthcare approaches for fall prevention (*n* = 17); (2) they did not include hospitalized or institutionalized older adults aged ≥ 65 years (*n* = 100); (3) they lacked relevant outcome measures (*n* = 7); and (4) they were not original research articles (*n* = 15). The study selection process is presented in a PRISMA flow diagram (Figure 1).

### 3.2. Characteristics of the Included Trials

Thirty-three studies were included in this review. For consistency, studies were categorized using a digital health approach rather than by the year of publication, as overlapping classifications could complicate interpretation. Of these, 20 studies evaluated fall detection systems, and 13 studies examined fall prediction models.

The included studies were conducted between 1993 and 2024 across multiple countries, including the United States (*n* = 7); Australia (*n* = 6); Germany (*n* = 4); Canada and France (*n* = 3 each); China (*n* = 2); and the United Kingdom, Switzerland, Turkey, Malaysia, Japan, Taiwan, and Belgium (*n* = 1 each). Regarding study design, five were RCTs, seven were quasi-experimental studies, 20 were observational studies, and one was a mixed-methods study. The study duration ranged from as short as one month to as long as five years, with 18 studies lasting less than one year.

The study settings were divided into hospitals (*n* = 17) and LTCFs (*n* = 16). In LTCF-related studies, various terms such as nursing homes, nursing facilities, and residential aged care facilities have been used; however, because these institutions were judged to be functionally similar, they were collectively interpreted under the category of LTCFs in this review.

Most participants were adults aged 80 years and older, although some studies reported age ranges instead of exact means. Common inclusion criteria included the ability to ambulate with or without assistance, preserved cognitive function, and a history of falls or fall risk. The participants were typically classified into high-risk categories based on their functional decline or frailty indicators. The detailed characteristics of the included studies are summarized in Table 2.

### 3.3. Quality Assessment

The methodological quality of the included studies was systematically evaluated according to study design (Supplementary S2).

Among the RCTs, domain-level assessments revealed heterogeneous risk patterns. Most RCTs showed some concerns related to the randomization process and deviations from intended interventions, while missing outcome data frequently contributed to a high risk. The measurement of outcomes and selective reporting varied among studies. Overall, four RCTs were judged to have a high risk of bias in at least one domain, and only one trial showed some concern across all domains, underscoring the need for more rigorously designed randomized studies on fall-related interventions (Supplementary S2 Figure S1).

The single mixed-method study demonstrated acceptable methodological quality in both the qualitative and quantitative components but showed limitations in integrating the two strands of evidence. Weaknesses include an unclear rationale for adopting a mixed-methods design, limited triangulation, and a small, non-representative sample. Overall, this study was judged to have moderate methodological quality, indicating acceptable internal coherence but limited generalizability (Supplementary S2 Table S1).

For diagnostic accuracy and experimental observational studies, the risk of bias assessment indicated moderate to high concerns across several domains. Patient selection and reference standards were the most frequent sources of bias due to small or convenience-based samples and reliance on observer-verified or report-based fall confirmation. The index test procedures were generally well described but were rarely blinded; additionally, the flow and timing were sometimes incomplete due to short follow-up or missing data. Collectively, these studies demonstrated an overall moderate risk of bias and low-to-moderate applicability, reflecting the exploratory and real-world nature of this research field (Supplementary S2 Table S2).

Prediction model studies showed a relatively low risk of bias in the participant and predictor domains, but a frequent high risk in the analysis domain. Common issues include small sample sizes, inadequate handling of missing data, and the absence of model calibration or overfitting assessments. The outcomes were generally well defined and objectively measured. Overall, the prediction model studies exhibited a moderate to high risk of bias, primarily driven by analytical limitations and a lack of external validation (Supplementary S2 Table S3).

Overall, although the included studies provided valuable evidence on digital healthcare approaches for fall detection and prevention, the methodological quality was generally modest, with frequent risks of bias. These limitations highlight the importance of cautious interpretation and underscore the need for well-designed, high-quality studies in this field.

### 3.4. Characteristics of Digital Approaches for Fall Detection and Prediction

#### 3.4.1. Fall Detection Systems

Twenty studies have investigated fall detection systems (Supplementary S3 Table S1). In hospital settings, the most frequently used systems are single-sensor devices, such as inertial measurement units (IMUs) [26,27,28,29,30,31] and pressure sensors embedded in mattresses or chairs [32,33,34,35]. More diverse systems have been adopted in LTCFs, including hybrid combinations of pressure mats, radar sensors, depth cameras, and vision-based monitoring [13,36,37,38,39,40,41,42,43,44]. IMUs are typically attached to the trunk or lower back to track mobility, while pressure or motion sensors are integrated into the environment (such as beds, chairs, or floors) to detect postural changes, transfers, or falls. Alerts were primarily directed at nursing staff in hospitals, whereas LTCF systems also notified caregivers and family members [13,36,38,39,40,41,44]. These systems allow for the continuous surveillance of residents with varying levels of mobility and dependency.

#### 3.4.2. Fall Prediction Models

Thirteen studies examined prediction models for fall prevention (Supplementary S3 Table S2). In hospitals, models mainly use sensor-derived gait data and electronic health records (EHRs) [12,45,46,47,48,49,50], whereas LTCF studies incorporate EHRs, minimum dataset records, or wearable sensor data [51,52,53,54,55,56]. Machine learning techniques, such as random forests, support vector machines, and gradient boosting, are the most common, whereas deep learning and regression-based approaches are less frequent. Across both settings, models that integrate multimodal predictors (sensor, clinical, and functional data) achieved higher discriminative performance than those using a single data type. Typically, machine learning and AI models outperform traditional fall risk screening tools in terms of accuracy and adaptability.

**Table 2:** Characteristics of included studies.

Authors (Year)	Country	Type of Facility	Study Design	Duration	Inclusion Criteria	Sample Size (I/C)	Mean Age (Range)
Fall Detection (*n* = 20)							
Can et al. (2024) [13]	Turkey	LTCF	Quasi-experimental	3 months	At high risk of falls Ambulatory Non-terminal	13/13	I: 82.7 ± 10.1 C: 81.9 ± 9.3
Dollard et al. (2022) [27]	Australia	Hospital	Mixed methods	23 months	65 years or older Not receiving palliative care	88	83.0 ± 9.0
Pham et al. (2022) [28]	Australia	Hospital	Quasi-experimental	5 years	65 years or older Admitted to the study wards	997/663	I: 85.3 ± 7.7 C: 85.8 ± 7.7
Saleh et al. (2021) [43]	France	LTCF	Prospective observational	13 months	80 years or older Ambulatory	16	80 years or older
Visvanathan et al. (2022) [30]	Australia	Hospital	RCT	2 years	65 years or older Admitted to the study wards	1244/1995	I: 84.0 ± 7.9 C: 81.9 ± 8.3
Borda et al. (2018) [37]	Australia	LTCF	Prospective observational	1 month	Requiring assistance and supervision during ambulation	4	87.0
White et al. (2018) [44]	USA	LTCF	Retrospective observational	5 months	65–95 years Complete fall and injury records	80/80	65–95 (range)
Gattinger et al. (2017) [39]	Switzerland	LTCF	RCT	11 months	With cognitive impairment With sleeping disorders	22/22	I: 86.4 ± 8.6 C: 88.7 ± 5.2
Shinmoto Torres et al. (2017) [29]	Australia	Hospital	Quasi-experimental	20–25 min	71 years or older Cognitively intact Ambulatory (independent or assisted) Admitted to a geriatric ward	26	71–93 (range)
Subermaniam et al. (2016) [34]	Malaysia	Hospital	Quasi-experimental	2 months	65 years or older Ambulatory (with or without aid)	31	83.0 ± 7.0
Lipsitz et al. (2016) [42]	USA	LTCF	Prospective observational	6 months	Fall history within 12 months Ability to comply with study requirements	62	86.2 ± 8.1
Abbate et al. (2014) [36]	Canada	LTCF	Prospective observational	1 month	75–92 years Diagnosed with Alzheimer’s disease Reisberg dementia stage 5–6 MMSE ≤ 12	4	75–92
Wong Shee et al. (2014) [33]	Australia	Hospital	Quasi-experimental	6 months	Clinically confirmed cognitive impairment At risk of falls during bed or chair exit	34	85.2 ± 7.7
Sahota et al. (2014) [32]	UK	Hospital	RCT	27 months	Admission to acute general ward with expected stay >24 h	918/921	84.6
Wolf et al. (2013) [31]	Germany	Hospital	RCT	13 months	STRATIFY score ≥3 Requiring assistance for movement	48/50	Not specified
Bloch et al. (2011) [26]	France	Hospital	Prospective observational	22 months	75 years or older Ambulatory or able to change posture to wear the sensor	10	83.4 ± 7.5
Capezuti et al. (2009) [38]	USA	LTCF	Prospective observational	8 months	65 years or older Capable of independent standing or ambulation Mild to moderate dementia without severe motor or acute medical issues	14	80.4 ± 5.9
Holmes et al. (2007) [40]	USA	LTCF	Quasi-experimental	15 months	Dementia unit residents ≥1 month Sensor attachment feasibility	38/70	I: 87.4 ± 7.0 C: 87.6 ± 7.5
Kelly et al. (2002) [41]	USA	LTCF	Quasi-experimental	5 months	At high risk of falls Able to wear a thigh-mounted patch	47	81.0 ± 7.0
Tideiksaar et al. (1993) [35]	USA	Hospital	RCT	9 months	Poor bed mobility on the POEMS scale	35/35	84 (67–97)
Fall Prediction (*n* = 13)							
Shao et al. (2024) [55]	China	LTCF	Prospective observational	6 months	60 years or older Resided in the facility for ≥1 month Ambulatory (independent or assisted)	864	84.0
Adeli et al. (2023) [12]	Canada	Hospital	Prospective observational	22 months	With dementia Able to walk independently at least 20 Minutes	54	76.4 ± 7.9
Millet et al. (2023) [48]	Spain	Hospital	Retrospective observational	5 years	65 years or older Prior fall history	304	80.3 ± 7.7
Boyce et al. (2022) [54]	USA	LTCF	Retrospective observational	6 years	Residents with ≥2 consecutive MDS assessments	3985	77.0
Chu et al. (2022) [47]	Taiwan	Hospital	Retrospective observational	2 years	Inpatients with HER and CGA records	1101	86.1
Song et al. (2022) [45]	China	Hospital	Prospective observational	Not stated	65 years or older Ambulatory Able to complete BBS No severe musculoskeletal, neurological gait disorders	48	LR: 72.3 ± 6.0 HR: 75.9 ± 6.9
Mehdizadeh et al. (2021) [50]	Canada	Hospital	Prospective observational	2 weeks +follow up 30 days	65 years or older With dementia Ambulatory (independent or assisted)	51	76.3 ± 7.9
Unger et al. (2021) [53]	Germany	LTCF	Prospective observational	1 year	Ambulatory (independent or assisted)	22	88.2 (78–99)
Buisseret et al. (2020) [51]	Belgium	LTCF	Prospective observational	6 months	65 years or older Able to complete TUG & 6MWT No severe musculoskeletal/ Cardiopulmonary conditions No significant cognitive impairment	73	83.0 ± 8.3
Suzuki et al. (2020) [56]	Japan	LTCF	Prospective observational	9 months	Diagnosed with Alzheimer’s disease No motor paresis Independent ambulation Able to perform KES	42	85.7 ± 5.6
Beauchet et al. (2018) [46]	France	Hospital	Prospective observational	6 months	Acute care admission Completion of nursing assessment within 24 h	848	83.0 ± 7.2
Gietzelt et al. (2014) [52]	Germany	LTCF	Prospective observational	11 months	65 years or older TUG ≥ 15 s MMSE < 24 points History of recurrent falls	40	76.0 ± 8.3
Marschollek et al. (2011) [49]	Germany	Hospital	Prospective observational	18 months	65 years or older Independent ambulation (TUG & 20 m walk) Adequate cognition for participation	46	81.3

Abbreviations: 6MWT: 6-min Walk Test; BBS: Berg Balance Scale; C: Control Group; CGA: Comprehensive Geriatric Assessment; EHR: Electronic Health Record; EMR: Electronic Medical Record; FAC: Functional Ambulation Category; HR: High-risk group; I: Intervention Group; KES: Knee Extension Strength; LR: Low-risk group; LTCF: Long-Term Care Facility; MDS: Minimum Data Set; MMSE: Mini-Mental State Examination; POEMS: Performance-Oriented Environmental Mobility Screen; RCT: Randomized Controlled Trial; STRATIFY: St. Thomas’s Risk Assessment Tool In Falling Elderly Inpatients; TUG: Timed Up and Go.

### 3.5. Effectiveness of Digital Approaches for Fall Prevention

#### 3.5.1. Fall Detection Systems

Nine studies evaluated the incidence of falls, reported either as absolute counts or standardized rates (Supplementary S4 Tables S1 and S2). Most hospital-based trials using pressure sensors reported no significant differences compared to controls [32,35]. In LTCFs, depth camera systems occasionally recorded no falls [37], while other trials even showed non-significant increases in fall events [44]. Only Holmes et al. [40] demonstrated a significant benefit, with an average monthly reduction of 1.6 falls (*p* = 0.017).

Four studies examined injurious falls (Supplementary S4 Table S3). Neither inertial nor pressure-sensor-based systems significantly reduced injury-related events. For example, Pham et al. [28] observed a nonsignificant reduction (aRR 0.56, 95% CI 0.17–1.79), whereas Sahota et al. [32] found a nonsignificant increase (aIRR 1.60, 95% CI 0.83–3.08). Holmes et al. [40] estimated the effects using regression coefficients and found no significant differences between the groups. Overall, the current evidence does not confirm that detection systems reduce total or injurious falls.

Five studies reported the number or proportion of participants who fell (Supplementary S4 Tables S4 and S5). Hospital-based trials ranged from fewer fallers in the intervention group (0 vs. 3, *p* = 0.243) [31] to nonsignificant increases in fall risk (OR 1.54, 95% CI 0.91–2.61) [30]. LTCF findings have been inconsistent, with some studies reporting temporary reductions [41] and others showing no effect [13,39].

The system performance was evaluated in ten studies (Supplementary S4 Table S6). The sensitivity ranged from 19% [42] to 100% [13,34,43], and the specificity ranged from 0.08% to 99.5%. False alarm rates vary widely, from negligible levels (<1 per day) to > 40% in certain pressure sensor systems [33].

Usability and acceptability were assessed in ten studies (Supplementary S4 Table S7). Patients, caregivers, and healthcare providers generally expressed high satisfaction (≥80–90%) and usability scores > 6.7/10), and many indicated a willingness to continue using it [27,29,34]. However, frequent false alarms and technical issues (e.g., sensor disconnection and battery problems) lead to alarm fatigue and reduced compliance. [40,44] Thus, although perceived as helpful, device-related limitations restrict its long-term use.

#### 3.5.2. Fall Prediction Models

Thirteen studies evaluated the prediction models, focusing on classification accuracy and key predictors (Supplementary S4 Tables S8 and S9).

##### Short-Term Prediction Models (<1 Month)

Three hospital-based studies assessed the short-term models. Adeli et al. [12] reported an AUROC of 0.76 using a multilayer perceptron (MLP), with a sensitivity of 72.8% and a specificity of 73.2%, based on cadence, estimated margin of stability (eMOS), gait scores, antipsychotic use, and the STRATIFY scale. Mehdizadeh et al. [50] achieved an AUROC of 0.80 with a Cox regression model, identifying fall history and eMOS as significant predictors. By contrast, Beauchet et al. [46] applied the NEAT model, which demonstrated high specificity (94.3%) but low sensitivity (29.6%), with key variables including age, disorientation, neuropsychiatric disorders, and prior home care services. These findings highlight moderate-to-high discrimination but show substantial heterogeneity across model types and predictors.

##### Long-Term Prediction Models (>3 Months)

Eight studies evaluated long-term prediction models. In hospitals, Millet et al. [48] reported good accuracy with a Random Forest bagging model (AUROC = 0.80; sensitivity = 70.0%; specificity = 80.5%) using BMI, weight, age, walking speed, and height as predictors. Chu et al. [47] applied XGBoost (AUROC = 0.73) with high sensitivity (91%) but low specificity (26%), based on the Braden score, ADL/IADL, age, and blood pressure.

In LTCFs, Shao et al. [55] achieved an AUROC of 0.75 (with a sensitivity of 85.2% and specificity of 61.8%) using a gradient boosting model that incorporated balance, grip strength, fatigue, fall history, and comorbidities. Boyce et al. [54] reported a lower accuracy (AUROC 0.67) using a hybrid CART logistic model that included age, psychotropic medication, fall history, mobility, and antidepressant use. Advanced approaches also showed variable performance: Unger et al. [53] achieved an AUROC of 0.89–0.96 and over 93% sensitivity with a gait-phase variability model, while Buisseret et al. [51] and Suzuki et al. [56] reported moderate accuracy (64–75%) using CNN- or decision tree-based methods with cognitive and functional predictors (MMSE, KES, FIM). Gietzelt et al. [52] reported an AUROC of 0.80, with a sensitivity of 78.2% and a specificity of 71.2%.

In all studies, machine learning and AI approaches generally outperformed conventional regression-based tools, particularly when combining multimodal predictors. Nonetheless, most studies have focused only on technical validation without assessing real-world clinical impact, leaving the practical effectiveness of the prediction models uncertain.

## 4. Discussion

### 4.1. Domains of Digital Approaches for Fall Detection and Prediction

This review synthesized 33 studies that evaluated digital healthcare approaches for fall prevention in older adults admitted to hospitals or residing in long-term LTCFs. These approaches are classified into two categories: detection-based systems (*n* = 20) and prediction-based models (*n* = 13). Detection systems are primarily designed to identify high-risk movements, such as bed exits or postural changes, and deliver real-time alerts to caregivers. Hospital-based studies have frequently implemented inertial or pressure sensors, with alerts directed mainly at nurses, reflecting the need for rapid responses in acute care settings. By contrast, LTCF studies have employed a broader variety of sensors aimed at covering larger environments and detecting subtle changes in mobility. Alarm recipients also differed across settings, with hospitals directing alerts mainly to professional nursing staff, whereas LTCFs often extended notifications to caregivers or family members, reflecting differences in organizational structures and staffing models. On the contrary, predictive models seek to estimate the likelihood of future falls by leveraging clinical, functional, and sensor-derived data. Hospitals tended to focus on short-term risk horizons (within one month), consistent with the heightened vulnerability of patients during acute admissions, whereas LTCFs evaluated medium- to long-term risks (three to six months) to guide ongoing supervision and care planning. Data sources also diverged; hospital-based studies frequently drew on EHR and admission assessments, whereas LTCFs relied more heavily on wearable sensors and longitudinal gait monitoring. These contextual differences underscore the fact that detection approaches are largely reactive, whereas prediction models aim to support anticipatory and individualized prevention strategies.

### 4.2. Comparative Effectiveness and Clinical Implications

#### 4.2.1. Fall Detection Systems

However, fall detection systems have not consistently reduced the incidence of falls or injuries from falls. Across outcomes such as fall count, fall rate, and proportion of fallers, no statistically significant improvements were demonstrated compared with the controls. In some cases, fall rates were higher in the intervention groups, a finding attributed to increased sensitivity and more complete recording of falls rather than actual increases in risk [19,57]. This finding underscores the fundamental limitations of the detection systems. They tended to improve monitoring and record keeping rather than directly preventing falls. In clinical settings, reliance on post-event detection has resulted in decreased enthusiasm for these systems, as proactive prevention is regarded as more valuable.

Performance outcomes varied substantially, with sensitivities ranging from 19% to 100% and specificities ranging from 0.3% to 99.5%. Pressure sensors often achieve high sensitivity (>90%) but produce false-positive rates of 30–40%. Multimodal systems, such as accelerometers combined with barometric sensors, show an improved balance between sensitivity and false alarms [43]. However, the performance benchmarks remain inconsistent, which limits their generalizability [58].

User acceptability and usability were generally favorable, with patients, caregivers, and healthcare staff reporting satisfaction rates above 80–90%. However, frequent false alarms contribute to alarm fatigue, a phenomenon recognized as a source of stress and cognitive overload among nurses [59]. Alarm fatigue reduces responsiveness to alerts and can compromise patient safety, underscoring the fact that technical accuracy alone is insufficient without considering workflow integration and staff burden. On balance, detection systems provide immediate safety benefits, particularly in high-acuity hospital wards; however, their long-term role in fall prevention is constrained and appears limited in routine practice given their inconsistent effectiveness and alarm fatigue [32]. The clinical use of such post-event detection technologies is gradually declining as healthcare practices increasingly prioritize proactive and preventive strategies.

#### 4.2.2. Fall Prediction Models

The fall prediction models demonstrated moderate to high discriminatory performance, with AUROC values ranging from 0.63 to 0.96. The sensitivity and specificity varied depending on the data source, model type, and sample characteristics. Short-term models often utilize the gait features of wearable sensors, whereas long-term models rely heavily on EHR data. Key predictors consistently associated with an elevated fall risk include age, prior falls, impaired mobility, the use of psychotropic or antidepressant medications, and functional measures such as ADL and IADL. The repeated identification of functional indicators is particularly notable because many conventional risk tools, such as the STRATIFY and Morse fall scales, do not directly capture ADL capacity. This finding suggests that functional assessments should be systematically incorporated into digital prediction tools for institutionalized older adults.

The model’s performance appeared to be influenced by the alignment between the data type and the algorithm. Gradient boosting and neural network models generally achieve relatively high accuracy for continuous gait variables [12,45,47], whereas regression-based or boosting approaches are effective for categorical EHR data [55]. Studies reporting AUROC values above 0.90 often struggled with low sensitivity due to class imbalance, highlighting the importance of an adequate sample size and balanced datasets [46]. These findings indicate that, although machine learning has technical potential, its clinical applicability remains uncertain without external validation and real-world testing.

Setting-specific differences are also observed. Hospital-based studies tend to focus on short-term fall prediction by leveraging admission assessments and acute functional measures. By contrast, LTCF-based studies emphasize long-term risks and often rely on longitudinal gait monitoring. Although both settings identified similar predictors, the relative contributions of the variables differed: gait-related metrics were more prominent in LTCFs, while demographic and clinical factors were more influential in hospitals.

The time horizon also influenced the model outcomes. Short-term models typically demonstrated an AUROC of around 0.76–0.80 with variable sensitivity (30–70%), while long-term models reported a wider AUROC range (0.73–0.96) and sensitivity of up to 90%. These variations likely reflect differences in data sources and event frequency, with short-term models limited by fewer fall events and long-term models influenced by broader functional decline trajectories.

Despite technically promising results, most prediction models have not yet been externally validated or implemented in clinical practice. Few studies have demonstrated how predicted risk scores translate into actionable care strategies, such as medication reviews, physiotherapy referrals, or enhanced supervision. Without integration and validation, predictive accuracy alone is insufficient to demonstrate real-world effectiveness or to improve patient outcomes.

### 4.3. Limitations

Several limitations of this review and the included studies should be acknowledged. The study designs were heterogeneous, comprising many quasi-experimental or observational studies and relatively few RCTs, which limited causal inferences. Outcomes were inconsistently reported, encompassing fall counts, fall rates, injurious falls, usability, and technical performance, which precluded a meta-analysis. Prediction model studies differ in terms of data sources, preprocessing strategies, and algorithm choices, making cross-study comparisons challenging. Most prediction models lack external validation and real-world testing, which limits their clinical applicability and generalizability. The nature of digital approaches complicates blinding and introduces potential performance and detection biases. While such characteristics may enhance real-world applicability, they also risk undermining internal validity. Moreover, several included studies exhibited a moderate to high risk of bias, which may have influenced the overall conclusions of this review. Studies with weaker methodological rigor often report larger or more inconsistent effects, suggesting that the apparent benefits of digital interventions should be interpreted with caution. Finally, the predominance of small-scale or single-center studies further constrains the robustness of the current evidence, underscoring the need for large-scale validation trials and implementation studies.

Additionally, publication bias could not be formally assessed because no quantitative synthesis was performed. Nevertheless, the predominance of small-scale studies and the absence of registered protocols suggest that publication and selective reporting biases cannot be excluded.

### 4.4. Clinical Implications and Future Directions

Despite these limitations, this study has several important implications. First, detection systems can provide immediate safety benefits but are unlikely to meaningfully reduce falls. Additionally, their role in routine practice remains limited, with mixed or negative trial findings and concerns regarding alarm fatigue [2,32,33,59]. Their future value lies in their integration with other strategies, with attention paid to minimizing false alarms and reducing alarm fatigue. Second, the prediction models hold substantial promise for proactive fall prevention. Essential predictors, such as age, prior falls, mobility, medication use, and ADL/IADL, should be systematically incorporated, and algorithms should be selected based on the data type to optimize performance. Third, contextual differences must be considered; hospitals may benefit from short-term prediction models that inform acute care planning, whereas LTCFs may require long-term models that capture functional decline and support ongoing care strategies. Finally, clinical adoption depends not only on technical accuracy but also on usability, workflow integration, and staff acceptance.

The future of fall prevention will likely depend on hybrid approaches that combine detection and prediction with evidence-based clinical interventions, such as physiotherapy, medication review, and environmental modification. Achieving this will require technological refinement, organizational commitment, external validation, and careful attention to patient-centered considerations including comfort, privacy, and usability. With these elements in place, digital healthcare approaches have the potential to shift fall prevention from a reactive to proactive strategy, ultimately enhancing the safety and quality of life for older adults in hospitals and LTCFs.

## 5. Conclusions

Digital healthcare approaches show promise for fall prevention in institutional settings; however, current evidence remains inconsistent. The refinement of detection systems, the external validation of prediction models, and their integration into patient-centered workflows are essential for achieving meaningful clinical benefits. Future studies should further evaluate the detection systems by addressing false alarms, improving clinical usability, and examining their long-term effectiveness.

## Figures and Tables

**Table 1:** Clinical Questions (Population, Intervention, Comparison, and Outcomes).

Category	Description
Population	Older adults (≥60 years) admitted to or residing in hospitals or LTCFs
Intervention	Digital healthcare approaches for fall detection and prevention (e.g., monitoring systems, sensors, ICT-based tools)
Comparison	Conventional strategies for fall detection and prevention without digital healthcare approaches
Outcomes	Fall-related outcomes (incidence, rate, injurious falls), device-related indicators (performance, cost), and user-centered outcomes (patient or caregiver satisfaction, usability, feasibility)

Abbreviations: LTCF, long-term care facilities; ICT, information and communication technology.

## Data Availability

The original contributions presented in this study are included in the article. Further inquiries can be directed to the corresponding authors.

## Supplementary Materials

The following supporting information can be downloaded at: https://www.mdpi.com/article/10.3390/medicina61111926/s1.
