# Supplementary material for: Digital Healthcare Approaches for Fall Detection and Prediction in Older Adults: A Systematic Review of Evidence from Hospital and Long-Term Care Settings
Source: Medicina (Kaunas). 2025 Oct 27;61(11):1926. doi: 10.3390/medicina61111926 (PMC12654721; doi:10.3390/medicina61111926)
Supplement: Supplementary file 1 [file medicina-61-01926-s001.zip › Supplementary S1.pdf]

Supplementary S1. Detailed search strategy

1. Ovid-Medline

1) (hospital or patient or "long term care" or "nursing home").ti,ab,kw.
2) (aged or older or elderly or geriatric or senior or senium or aging).ti,ab,kw.
3) (fall or "accidental falls" or falling).ti,ab,kw.
4) (accelerometer or alarm or "ambulatory monitoring" or "artificial intelligence" or camera or detection or "digital health" or "electronic device" or gyroscope or ICT or Kinect or motion or pad or "radio wave" or robot or sensor or "smart health" or vibration or wearable).ti,ab,kw.
5) 1 and 2 and 3 and 4

2. Embase

1) ('hospital'/exp OR 'hospital':ti,ab,kw OR 'patient'/exp OR 'patient':ti,ab,kw OR 'long term care'/exp OR 'long term care':ti,ab,kw OR 'nursing home'/exp OR 'nursing home':ti,ab,kw)
2) ('aged'/exp OR 'aged':ti,ab,kw OR 'older adults'/exp OR 'older adults':ti,ab,kw OR 'geriatric'/exp OR 'geriatric':ti,ab,kw OR 'senior':ti,ab,kw OR 'elderly':ti,ab,kw OR 'senium':ti,ab,kw OR 'aging'/exp OR 'aging':ti,ab,kw)
3) ('falling'/exp OR 'fall':ti,ab,kw OR 'accidental falls':ti,ab,kw OR 'falling':ti,ab,kw)
4) ('accelerometer'/exp OR 'accelerometer':ti,ab,kw OR 'alarm':ti,ab,kw OR 'ambulatory monitoring'/exp OR 'ambulatory monitoring':ti,ab,kw OR 'artificial intelligence'/exp OR 'artificial intelligence':ti,ab,kw OR 'camera'/exp OR 'camera':ti,ab,kw OR 'detection'/exp OR 'detection':ti,ab,kw OR 'digital health'/exp OR 'digital health':ti,ab,kw OR 'electronic device'/exp OR 'electronic device':ti,ab,kw OR 'gyroscope'/exp OR 'gyroscope':ti,ab,kw OR 'ict'/exp OR 'ict':ti,ab,kw OR 'kinect':ti,ab,kw OR 'motion'/exp OR 'motion':ti,ab,kw OR 'pad':ti,ab,kw OR 'radio wave':ti,ab,kw OR 'robot'/exp OR 'robot':ti,ab,kw OR 'sensor'/exp OR 'sensor':ti,ab,kw OR 'smart health':ti,ab,kw OR 'vibration':ti,ab,kw OR 'wearable':ti,ab,kw)
5) #1 AND #2 AND #3 AND #4

3. Cochrane library

1) MeSH descriptor: [Hospital] explode all trees
2) MeSH descriptor: [Patient] explode all trees
3) MeSH descriptor: [Long-Term Care] explode all trees
4) MeSH descriptor: [Nursing Homes] explode all trees
5) #1 OR #2 OR #3 OR #4
6) MeSH descriptor: [Aged] explode all trees

7) (older OR elderly OR geriatric OR senior OR senium OR aging):ti,ab,kw.

8) #6 OR #7

9) MeSH descriptor: [Falls] explode all trees

10) (fall OR accidental falls OR falling):ti,ab,kw.

11) #9 OR #10

12) (accelerometer OR alarm OR “ambulatory monitoring” OR “artificial intelligence” OR camera OR detection OR “digital health” OR “electronic device” OR gyroscope OR ICT OR Kinect OR motion OR pad OR “radio wave” OR robot OR sensor OR “smart health” OR vibration OR wearable):ti,ab,kw.

13) #5 AND #8 AND #11 AND #12

4. CINAHL (EBSCOhost)

1) (MH "Hospital") OR (MH "Patients") OR (MH "Long Term Care") OR (MH "Nursing Homes")

2) (MH "Aged+") OR (TI aged OR TI older OR TI elderly OR TI geriatric OR TI senior OR TI senium OR TI aging OR AB aged OR AB older OR AB elderly OR AB geriatric OR AB senior OR AB senium OR AB aging)

3) (MH "Accidental Falls") OR (TI fall OR TI "accidental falls" OR TI falling OR AB fall OR AB "accidental falls" OR AB falling)

4) (MH "Accelerometers") OR (MH "Artificial Intelligence") OR (MH "Ambulatory Monitoring") OR (MH "Digital Health") OR (MH "Sensors") OR (MH "Robotics") OR (MH "Wearable Electronic Devices") OR (TI accelerometer OR TI alarm OR TI "ambulatory monitoring" OR TI "artificial intelligence" OR TI camera OR TI detection OR TI "digital health" OR TI "electronic device" OR TI gyroscope OR TI ICT OR TI Kinect OR TI motion OR TI pad OR TI "radio wave" OR TI robot OR TI sensor OR TI "smart health" OR TI vibration OR TI wearable OR AB accelerometer OR AB alarm OR AB "ambulatory monitoring" OR AB "artificial intelligence" OR AB camera OR AB detection OR AB "digital health" OR AB "electronic device" OR AB gyroscope OR AB ICT OR AB Kinect OR AB motion OR AB pad OR AB "radio wave" OR AB robot OR AB sensor OR AB "smart health" OR AB vibration OR AB wearable)

5) #1 AND #2 AND #3 AND #4

5. PubMed

1) (hospital OR patient OR "long term care" OR "nursing home")

2) (aged OR older OR elderly OR geriatric OR senior OR senium OR aging OR aged[MeSH Terms])

3) #1 AND #2

4) (fall OR "accidental falls" OR falling OR "Accidental Falls"[MeSH Terms])

5) (accelerometer OR alarm OR "ambulatory monitoring" OR "artificial intelligence" OR camera OR detection OR "digital health" OR "electronic device" OR gyroscope OR ICT OR Kinect OR motion OR pad OR "radio wave" OR robot OR sensor OR "smart health" OR vibration OR wearable OR "Artificial Intelligence"[MeSH Terms])

6) #3 AND #4 AND #5

6. IEEE Xplore

1) ("All Metadata":hospital) OR ("All Metadata":patient) OR ("All Metadata":"long term care") OR ("All Metadata":"nursing home")

2) ("All Metadata":aged) OR ("All Metadata":older) OR ("All Metadata":elderly) OR ("All Metadata":geriatric) OR ("All Metadata":senior) OR ("All Metadata":senium) OR ("All Metadata":aging)

3) ("All Metadata":fall) OR ("All Metadata":"accidental falls") OR ("All Metadata":falling)

4) ("All Metadata":accelerometer) OR ("All Metadata":alarm) OR ("All Metadata":"ambulatory monitoring") OR ("All Metadata":"artificial intelligence") OR ("All Metadata":camera) OR ("All Metadata":detection) OR ("All Metadata":"digital health") OR ("All Metadata":"electronic device") OR ("All Metadata":gyroscope) OR ("All Metadata":ICT) OR ("All Metadata":Kinect) OR ("All Metadata":motion) OR ("All Metadata":pad) OR ("All Metadata":"radio wave") OR ("All Metadata":robot) OR ("All Metadata":sensor) OR ("All Metadata":"smart health") OR ("All Metadata":vibration) OR ("All Metadata":wearable)

5) #1 AND #2 AND #3 AND #4

7. RISS

1) (hospital | patient | "long term care" | "nursing home")

2) (aged | older | elderly | geriatric | senior | senium | aging)

3) (fall | "accidental falls" | falling)

4) (accelerometer | alarm | "ambulatory monitoring" | "artificial intelligence" | camera | detection | "digital health" | "electronic device" | gyroscope | ICT | Kinect | motion | pad | "radio wave" | robot | sensor | "smart health" | vibration | wearable)

8. KoreaMed

1) (hospital[ALL] OR patient[ALL] OR "long term care"[ALL] OR "nursing home"[ALL])

2) (aged[ALL] OR older[ALL] OR elderly[ALL] OR geriatric[ALL] OR senior[ALL] OR senium[ALL] OR aging[ALL])

3) (fall[ALL] OR "accidental falls"[ALL] OR falling[ALL])

4) (accelerometer[ALL] OR alarm[ALL] OR "ambulatory monitoring"[ALL] OR "artificial intelligence"[ALL] OR camera[ALL] OR detection[ALL] OR "digital health"[ALL] OR "electronic device"[ALL] OR gyroscope[ALL] OR ICT[ALL] OR Kinect[ALL] OR motion[ALL] OR pad[ALL] OR "radio wave"[ALL] OR robot[ALL] OR sensor[ALL] OR "smart health"[ALL] OR vibration[ALL] OR wearable[ALL])

5) #1 AND #2 AND #3 AND #4

9. KMBase

1) ((hospital|title) OR (patient|title) OR ("long term care"|title) OR ("nursing home"|title))

2) ((aged|title) OR (older|title) OR (elderly|title) OR (geriatric|title) OR (senior|title) OR (senium|title) OR (aging|title))

3) ((fall|title) OR ("accidental falls"|title) OR (falling|title))

4) ((accelerometer|title) OR (alarm|title) OR ("ambulatory monitoring"|title) OR ("artificial intelligence"|title) OR (camera|title) OR (detection|title) OR ("digital health"|title) OR ("electronic device"|title) OR (gyroscope|title) OR (ICT|title) OR (Kinect|title) OR (motion|title) OR (pad|title) OR ("radio wave"|title) OR (robot|title) OR (sensor|title) OR ("smart health"|title) OR (vibration|title) OR (wearable|title))

5) ((hospital|abstract) OR (patient|abstract) OR ("long term care"|abstract) OR ("nursing home"|abstract))

6) ((aged|abstract) OR (older|abstract) OR (elderly|abstract) OR (geriatric|abstract) OR (senior|abstract) OR (senium|abstract) OR (aging|abstract))

7) ((fall|abstract) OR ("accidental falls"|abstract) OR (falling|abstract))

8) ((accelerometer|abstract) OR (alarm|abstract) OR ("ambulatory monitoring"|abstract) OR ("artificial intelligence"|abstract) OR (camera|abstract) OR (detection|abstract) OR ("digital health"|abstract) OR ("electronic device"|abstract) OR (gyroscope|abstract) OR (ICT|abstract) OR (Kinect|abstract) OR (motion|abstract) OR (pad|abstract) OR ("radio wave"|abstract) OR (robot|abstract) OR (sensor|abstract) OR ("smart health"|abstract) OR (vibration|abstract) OR (wearable|abstract))

9) (#1 AND #2 AND #3 AND #4) OR (#5 AND #6 AND #7 AND #8)

10. KISS

1) ("hospital"[All Fields] OR "patient"[All Fields] OR "long term care"[All Fields] OR "nursing home"[All Fields])

2) ("aged"[All Fields] OR "older"[All Fields] OR "elderly"[All Fields] OR "geriatric"[All Fields] OR "senior"[All Fields] OR "senium"[All Fields] OR "aging"[All Fields])

3) ("fall"[All Fields] OR "accidental falls"[All Fields] OR "falling"[All Fields])

4) ("accelerometer"[All Fields] OR "alarm"[All Fields] OR "ambulatory monitoring"[All Fields] OR "artificial intelligence"[All Fields] OR "camera"[All Fields] OR "detection"[All Fields] OR "digital health"[All Fields] OR "electronic device"[All Fields] OR "gyroscope"[All Fields] OR "ICT"[All Fields] OR "Kinect"[All Fields] OR "motion"[All Fields] OR "pad"[All Fields] OR "radio wave"[All Fields] OR "robot"[All Fields] OR "sensor"[All Fields] OR "smart health"[All Fields] OR "vibration"[All Fields] OR "wearable"[All Fields])

5) #1 AND #2 AND #3 AND #4
