# Supplementary material for: Digital Healthcare Approaches for Fall Detection and Prediction in Older Adults: A Systematic Review of Evidence from Hospital and Long-Term Care Settings
Source: Medicina (Kaunas). 2025 Oct 27;61(11):1926. doi: 10.3390/medicina61111926 (PMC12654721; doi:10.3390/medicina61111926)
Supplement: Supplementary file 1 [file medicina-61-01926-s001.zip › Supplementary S2.pdf]

Supplementary S2 Quality assessment

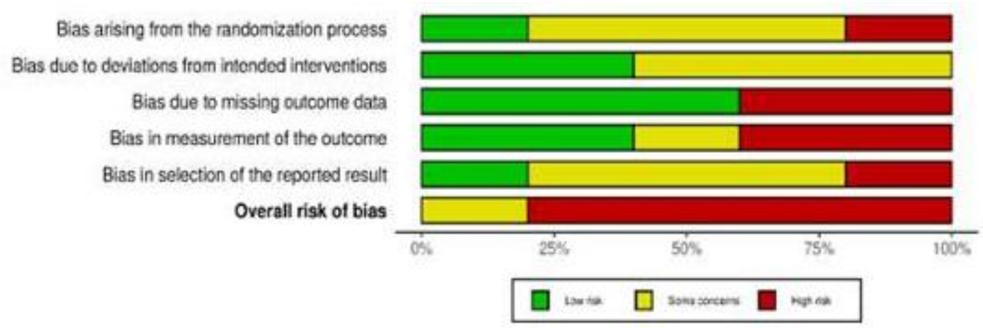

(a)

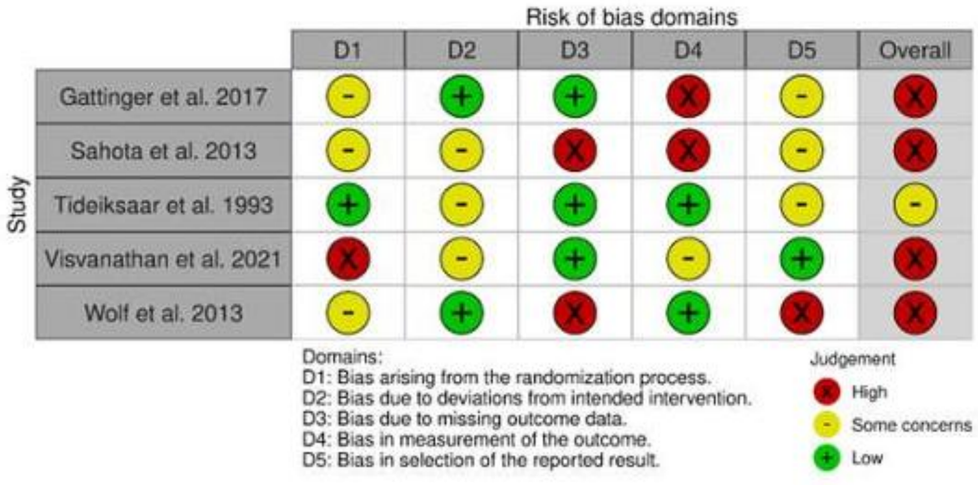

(b)

S2 Figure S1. Risk of bias in digital approaches for fall detection and prevention among older adults in healthcare facilities (a) Risk of Bias Graph (b) Risk of Bias Summary

S2 Table S1. Results of the risk of bias assessment conducted using the Quality Assessment of Diagnostic Accuracy Studies-2 (QUADAS-2) tool

Author (year)	Diagnostic studies				
	Patient Selection	Index Test	Reference Standard	Flow & Timing	Overall Risk
Can et al. (2024)	Moderate	Low	Moderate	Moderate	Moderate
Pham et al. (2022)	Moderate	Low	Moderate	Moderate	Moderate
Saleh et al. (2021)	Low	Moderate	Low	Moderate	Moderate
Borda et al. (2018)	High	Moderate	High	High	High
White et al. (2018)	Moderate	Moderate	Moderate	High	High
Shinmoto et al. (2017)	Low	Low	Low	Moderate	Moderate
Subermaniam et al. (2017)	Moderate	Moderate	Moderate	Low	Moderate
Lipsitz et al. (2016)	Moderate	Low	Moderate	Moderate	Moderate
Abbate et al. (2014)	High	Moderate	High	Moderate	High
Wong Shee et al. (2014)	Moderate	Moderate	High	Moderate	High
Bloch et al. (2011)	High	Moderate	Moderate	High	High
Capezuti et al. (2009)	Moderate	Low	Moderate	Moderate	Moderate
Holmes et al. (2007)	Moderate	Low	High	Moderate	High
Kelly et al. (2002)	Moderate	Moderate	High	Moderate	High

S2 Table S2. Results of the risk of bias assessment conducted using the Mixed Methods Appraisal Tool (MMAT)

Author (year)	Diagnostic studies					
	Research questions clear and relevant to data	Adequacy of qualitative data collection	Appropriateness of quantitative design and analysis	Integration of qual & quant components	Consideration of limitations and bias	Overall quality
Dollard et al. (2022)	Yes	Yes	Yes	Moderate	Yes	Moderate

S2 Table S3. Results of the risk of bias assessment conducted using the Prediction model Risk of Bias Assessment Tool (PROBAST)

**Author (year)**	**Cohort studies**				
	**Participants**	**Predictors**	**Outcome**	**Analysis**	**Overall Risk**
Shao et al. (2024)	Low	Low	Low	Moderate	Moderate
Adeli et al. (2023)	Low	Moderate	Low	Moderate	Moderate
Millet et al. (2023)	Moderate	Moderate	Low	High	High
Boyce et al. (2022)	Low	Moderate	Low	Moderate	Moderate
Chu et al. (2022)	Moderate	Low	Low	High	High
Song et al. (2022)	High	Moderate	Low	Moderate	High
Mehdizadeh et al. (2021)	Low	Moderate	Low	Moderate	Moderate
Unger et al. (2021)	Moderate	Low	Low	High	High
Buisseret et al. (2020)	Moderate	Moderate	Low	High	High
Suzuki et al (2020)	Moderate	Low	Low	High	High
Beauchet et al. (2018)	Moderate	Moderate	Low	Moderate	Moderate
Gietzelt et al. (2014)	Moderate	Moderate	Low	Moderate	Moderate
Marschollek et al. (2011)	Moderate	Low	Low	Moderate	Moderate
