# Supplementary material for: Digital Healthcare Approaches for Fall Detection and Prediction in Older Adults: A Systematic Review of Evidence from Hospital and Long-Term Care Settings
Source: Medicina (Kaunas). 2025 Oct 27;61(11):1926. doi: 10.3390/medicina61111926 (PMC12654721; doi:10.3390/medicina61111926)
Supplement: Supplementary file 1 [file medicina-61-01926-s001.zip › Supplementary S3.pdf]

Supplementary S3 Characteristics of digital healthcare approaches for fall detection and prevention

S3 Table S1. Characteristics of fall detection systems

Tech	Authors (year)	Target	Location	Alert recipient	Outcome
Hospitals					
IMU	Dollard et al. (2022)	Specific movement	Sternum	Nurse (mobile app)	• Acceptability
	Pham et al. (2022)	Specific movement	Sternum	Nurse (mobile app)	• Injurious fall rate<br>• Cost-effectiveness
	Visvanathan et al. (2021)	Specific movement	Sternum	Nurse (mobile app)	• Fall rate<br>• Injurious fall rate<br>• Proportion of fallers
	Shnmoto Torres et al. (2017)	Specific movement	Sternum	Nurse (station)	• Performance<br>• Acceptability
	Wolf et al. (2013)	Specific movement	Thigh	Nurse (station)	• Number of fallers<br>• Acceptability
	Bloch et al. (2011)	Fall-like movement	Waist	Patient (sound)	• Performance<br>• Acceptability
Pressure	Subermaniam et al. (2017)	Pressure loss	Mattress	Nurse (station, paper, room alarm)	• Performance<br>• Acceptability<br>• Workload reduction
	Shee et al. (2014)	Pressure loss	Mattress, Chair	Nurse (station, paper, room alarm)	• Fall rate<br>• Performance<br>• Feasibility<br>• Acceptability
	Sahota et al. (2013)	Pressure loss	Mattress, Chair	Nurse (paper)	• Number of falls<br>• Injurious fall rate
	Tideiksaar et al. (1993)	Pressure loss	Mattress	Nurse (station)	• Number of falls<br>• Performance<br>• Acceptability
Long-term care facilities					
Rader	Can et al. (2024)	Specific movement	Walls, Ceiling	Nurse (station, app, room alarm)	• Number of falls<br>• Performance
IMU Barometric	Saleh et al. (2021)	Fall-like movement	Neck or wrist	None	• Performance
Depth camera	Borda et al. (2018)	Fall-like movement	Walls, Ceiling	None	• Number of falls<br>• Feasibility<br>• Acceptability
Not specified	White et al. (2018)	Specific movement	Bed	Nurse (station)	• Number of falls<br>• Number of injurious falls
Pressure	Gattinger et al. (2017)	Pressure distribution	Mattress	Nurse (station)	• Proportion of fallers
IMU Electode	Abbate et al. (2014)	Fall-like movement	Wrist, Hand	Nurse (station, paper) Caregiver (app)	• Usability<br>• Acceptability
Pressure IR	Capezuti et al. (2009)	Pressure loss Specific movement	Mattress, Bedside	Nurse (station, room alarm)	• Performance
Presssure Contact	Holmes et al. (2007)	Pressure loss Specific movement	Mattress, Door frame	Nurse (paper)	• Number of falls<br>• Injurious fall rate
IMU	Lipsitz et al. (2006)	Fall-like movement	Neck	None	• Performance

Tilt	kelly et al. (2002)	Fall-like movement	Thigh	Nurse (station, app, room alarm)	• Number of fallers<br>• Fall rate<br>• Proportion of falls

Abbreviations: app: Application; IR: Infrared Rays; IMU: Inertial Measurement Unit

S3 Table S2. Characteristics of fall prediction models

Author (Year)	Collected data	Fall Prediction Target	Fall data source	Best Performing Model
Hospital				
Adeli et al. (2023)	• Sensor-derived Gait features<br>• Clinical data	4-week	EMR, Report	MLP (only use)
Millet et al. (2023)	• HER data	Recurrent	EHR	Bagging with RF
Chu et al. (2022)	• Admission EHR and CGA data	During hospitalization	CGA	XGBoost
Song et al. (2002)	• Sensor-derived gait features	Faller classification	Classified by BBS score	Decision Tree, GBDT, AdaBoost
Mehdizadeh et al. (2021)	• Sensor-derived gait features<br>• EHR data<br>• Clinician-assessed scales	7-day<br>30-day	EHR, Report Interview	Statistical only (Cox regression)
Beauchet et al. (2018)	• Admission nurses' initial assessments	21-day	EMR, Report	NEAT
Marschollek et al. (2011)	• Sensor-derived motion data<br>• Clinical evaluation variable	1 year post-discharge	EMR, Report	Simple Logistic
Long-term care facilities				
Shao et al. (2024)	• EHR data	6-month	EMR, Report	GBM
Boyce et al. (2022)	• MDS data	90-day	MDS	Hybrid (CART + LR)
Unger et al. (2021)	• Sensor-derived gait features	6 months pre/post gait monitoring	Report	Statistical only
Busseret et al. (2020)	• Sensor-derived gait features	6-months	Report	CNN-based AI (only use)
Suzuki et al. (2020)	• Age, MMSE, KES, FIM	Time to fall (150d/300d /No fall)	Report	CNN (only use)
Gietzelt et al. (2014)	• Sensor-derived gait features	Time to fall (2/4/8 mon)	Report	Decision Tree (only use)

Abbreviations: AdaBoost: Adaptive Boosting; CART: Classification and Regression Tree; CGA:Comprehensive Geriatric Assessment; CNN: Convolutional Neural Network; D: Day(s); DL: Deep Learning; EHR: Electronic Health Record; EMR: Electronic Medical Record; GBDT Gradient Boosting Decision Tree; GBM: Gradient Boosting Machine; KES: Knee Extension Strength; LR: Logistic Regression; ML: Machine Learning; MLP: Muiti-Layer Perceptron; MMSE: Mini-Mental State Examination; Mon: Month(s) NEAT: NeuroEvolution of Augmenting Topologies; NPI: Neuropsychiatric Inventory; RF: Random Forest; STRATIFY: St. Thomas's Risk Assessment Tool In Falling Elderly Inpatents; XGBoost: Extreme Gradient Boosting
