# Supplementary material for: Digital Healthcare Approaches for Fall Detection and Prediction in Older Adults: A Systematic Review of Evidence from Hospital and Long-Term Care Settings
Source: Medicina (Kaunas). 2025 Oct 27;61(11):1926. doi: 10.3390/medicina61111926 (PMC12654721; doi:10.3390/medicina61111926)
Supplement: Supplementary file 1 [file medicina-61-01926-s001.zip › Supplementary S4.pdf]

Supplementary S4 Fall-related outcomes of digital healthcare approaches for fall detection and prevention

S4 Table S1. Fall-related outcomes of fall detection systems: number of falls

Facility	Author (year)	Sensor type	Location	Outcome
Hospital	Sahota et al. (2013)	Pressure	Mattress, Chair	aIRR: 0.90 (95% CI: 0.66–1.22; p = 0.50)
	Tideiksaar et al. (1993)	Pressure	Mattress	IG/CG: 1/4 falls (p = 1.00)
Long-term care facilities	Borda et al. (2018)	Depth camera	Walls, Ceiling	0 falls recorded
	White et al. (2018)	Not specified	Bed	IG:18.8 ± 1.92 vs CG: 14.0 ± 2.55 (MD: +4.8)
	Kelly et al. (2002)	Tilt	Thigh	Pre: 11 / During: 1 / Post: 17

Abbreviations: aIRR: Adjusted Incidence Rate Ratio; CG: Control Group; CI: Confidence Interval; I: Intervention Group; MD: Mean Difference

S4 Table S2. Fall-related outcomes of fall detection systems: fall rates

Facility	Author (year)	Sensor type	Location	Outcome
Hospital	Visvanathan et al. (2021)	Inertial	Sternum	aRR: 1.41 (95% CI 0.85–2.34; p = 0.19)
	Wong Shee et al. (2014)	Pressure	Mattress, Chair	IRR: 1.92, p = 0.01
Long-term care facilities	Kelly et al. (2002)	Tilt	Thigh	Pre 4.0 / During 0.3 / Post 3.4 (per 100 patient-days)

Abbreviations: aIRR: Adjusted Incidence Rate Ratio; CG: Control Group; CI: Confidence Interval; I: Intervention Group; MD: Mean Difference

S4 Table S3. Fall-related outcomes of fall detection systems: injurious fall rates

Facility	Author (year)	Sensor type	Location	Outcome
Hospital	Pham et al. (2022)	Inertial	Sternum	aRR: 0.56 (95% CI 0.17-1.79)
	Visvanathan et al. (2021)	Inertial	Sternum	aRR: 0.90 (95% CI 0.38–2.14; p = 0.81)
	Sahota et al. (2013)	Pressure	Mattress, Chair	aIRR: 1.60 (95% CI 0.83-3.08; p = 0.15)

Abbreviations: aIRR: Adjusted Incidence Rate Ratio; aRR: Adjusted Rate Ratio; CI: Confidence Interval

S4 Table S4. Fall-related outcomes of fall detection systems: number of fallers

Facility	Author (year)	Sensor type	Location	Outcome
Hospital	Wolf et al. (2013)	Inertial	Thigh	IG: 0/48, CG: 3/50 (p = 0.24)
Long-term care facilities	Can et al. (2024)	Rader	Walls, Ceiling	IG: 1/13, CG: 2/13 (p = 0.54)

Abbreviations: CG: Control Group; I: Intervention Group

S4 Table S5. Fall-related outcomes of fall detection systems: number of fallers

Facility	Author (year)	Sensor type	Location	Outcome
Hospital	Visvanathan et al. (2021)	Inertial	Sternum	OR: 1.54 (95% CI 0.91-2.61; p = 0.11)
Long-term care facilities	Gattinger et al. (2017)	Depth camera	Walls, Ceiling	IG: 10%→20%<br>CG: 10%→30%→20%<br>p = 0.70
	Kelly et al. (2002)	Tilt	Thigh	Pre: 15.2% / During: 2.1% / Post: 22.5%

Abbreviations: CG: Control Group; CI: Confidence Interval; IG: Intervention Group; Int: Interaction; OR: Odd Ratio

S4 Table S6. Fall-related outcomes of fall detection systems: performance

Facility	Author (year)	Sensor type	Location	Outcome
Hospital	Shinmoto Torres et al. (2017)	Inertial	Sternum	• Sensitivity: 81.4%
	Subermaniam et al. (2017)	Pressure	Mattress	• Sensitivity: 100%<br>• PPV 68%; FPR 31%.
	Wong Shee et al. (2014)	Pressure	Mattress, Chair	• Sensitivity: 94.8%<br>• False alarm rate: 42.8%
	Bloch et al. (2011)	Inertial	Waist	• Sensitivity: 62.5%<br>• Specificity: 99.5%,<br>• PPV: 16.7%, NPV: 99.9%
	Tideiksaar et al. (1993)	Pressure	Mattress	• TP/FP: 120/23<br>• PPV: 84%
Long-term care facilities	Can et al. (2024)	Rader	Walls, Ceiling	• Sensitivity:100%<br>• PPV: 68%<br>• False alarm: 0.22/day:
	Saleh et al. (2021)	Inertial, Barometric	Neck or Wrist	• Sensitivity: 100%<br>• Specificity: 98.5%(neck), 96.7%(wrist)<br>• FP: 1 per 25days (neck)<br>1 per 3days (wrist)
	Lipstiz et al. (2016)	Inertial	Neck	• Sensitivity: 19%<br>• Specificity: 8.3%<br>• PPV 13%, FPR 87%
	Capezuti et al. (2009)	Pressure, IR	Mattress, Bedside	***Pressure only alarm***<br>• Sensitivity: 11 %<br>• Specificity: 0.08 %<br>***Dual sensor alarm***<br>• Sensitivity: 71%<br>• Specificity: 0.3 %
	Kelly et al. (2002)	Tilt	Thigh	False alarms: none

Abbreviations: FP: False Positive; FPR: False Positive Rate; NPV : Negative Predictive Value; PPV: Positive Predictive Value; TP: True Positive;

S4 Table S7. Fall-related outcomes of fall detection systems: acceptability, feasibility

Facility	Author (year)	Sensor type	Location	Outcome
Hospital	Dollard et al. (2022)	Inertial	Sternum	• Acceptance: 89% (patients)<br>91% (nurses)<br>• Ease of use: 81%
	Shinmoto Torres et al. (2017)	Inertial	Sternum	• Usability score: 7.83 → 9.03<br>• All domains ≥ 6.7 (PA, Anxiety, Equip. Privacy)
	Subermaniam et al. (2017)	Pressure	Mattress	• Easy: 97%<br>• Willingness: 87%<br>• Useful: 83%
	Wong Shee et al. (2014)	Pressure	Mattress, Chair	• High satisfaction<br>• Needs alarm setting improvement
	Wolf et al. (2013)	Inertial	Thigh	• High acceptance (patients, nurses)<br>• Minimal false alarms
	Bloch et al. (2011)	Inertial	Waist	• Acceptability "excellence" (7/8)
	Tideiksaar et al. (1993)	Pressure	Mattress	• High acceptance (patients, family, staff)
Long-term care facilities	Borda et al. (2018)	Depth camera	Walls, Ceiling	• 24/7 operation,<br>• sensor/network/power issues
	Abbate et al. (2014)	Inertial Electode	Waist, Head	• System (waist): High by D3<br>• System (head): Moderate after redesign
	Kelly et al. (2002)	Tilt	Thigh	• High acceptance (patients, nurses)

Abbreviations: D: Day; Equip.: Equipment; PA: Physical activity;

S4 Table S8. Fall-related outcomes of fall prediction models: short-term predictive performance

Facility	Author (year)	Best model	Outcome	Main predictors
Hospital	Adeli et al. (2023)	MLP (only use)	• AUROC: 0.76<br>• Sen: 72.8%<br>• Spe: 73.2%	***Best combination***<br>• Cadence<br>• eMOS<br>• UPDRS gait (initial & recent avg)<br>• Antipsychotics<br>• STRATIFY
	Mehdizadeh et al. (2021)	Statistical only (Cox regression)	• AUROC: 0.80<br>• Sen: NR<br>• Spe: NR	• Fall history<br>(HR = 6.15, 95% CI: 2.15–17.56, p=.006)<br>• eMOS<br>(HR=0.60, 95% CI: 0.44-0.82, p=0.001)
	Beauchet et al. (2018)	NEAT	• AUROC: NR<br>• Sen: 29.6%<br>• Spe: 94.3%	• Age (HR 1.04, p = 0.011)<br>• Disorietation<br>(HR > 2.00, p < 0.003)<br>• Neuropsychiatric disorders<br>(HR 2.34, p < 0.001)<br>• Use of formal home care services prior to admission<br>(HR 2.37, p=.022)

Abbreviations: AUROC: Area Under the Receiver Operating Characteristic Curve; CI: Confidence Interval; eMOS: Estimated Margin of Stability; HR: Hazard Ratio; MLP: Multi-Layer Perceptron; NR: Not Reported; Sen: Sensitivity; Spe: Specificity; STRATIFY: St. Thomas's Risk Assessment Tool In Falling Elderly Inpatients; UPDRS: Unified Parkinson's Disease Rating Scale

S4 Table S9. Fall-related outcomes of fall prediction models: long-term predictive performance

<table>
<tr><th>Facility</th><th>Author (year)</th><th>Best model</th><th>Outcome</th><th>Main predictors</th></tr>
<tr><td rowspan="2">Hospital</td><td>Millet et al. (2023)</td><td>Bagging with RF</td><td>AUROC: 0.803
Sen: 70.0%
Spe: 80.5%</td><td>• BMI
• Weight
• Age
• Walking speed
• Height (ranked)</td></tr>
<tr><td>Chu et al. (2022)</td><td>XGBoost</td><td>• AUROC: 0.73
• Sen: 91%
• Spe: 26%</td><td>• ADL
• Braden score
• ADL
• Age
• SBP (ranked)</td></tr>
<tr><td rowspan="7">Long-term care facilities</td><td>Shao et al. (2024)</td><td>GBM</td><td>• AUROC: 0.75
• Sen: 85.2%
• Spe: 61.8%</td><td>• Balance
• Grip strength
• Fatigue
• Fall history
• Age
• Comorbidity (ranked)</td></tr>
<tr><td>Boyce et al. (2022)</td><td>Hybrid CART-LR</td><td>• AUROC: 0.67
• Sen: 57%
• Spe: 69%</td><td>• Age
• Psychotropics
• Fall history
• Mobility
• Walking aid
• Antidepressants
• Behavior
(recent change, aggression toward others)</td></tr>
<tr><td rowspan="2">Unger et al. (2021)</td><td rowspan="2">Statistical only</td><td>• AUROC: 0.96
• Sen: 93.3%
• Spe: NR</td><td>• Stance/swing phase variability</td></tr>
<tr><td>• AUROC: 0.89
• Sen: 93.3%
• Spe: NR</td><td>• Double support time</td></tr>
<tr><td>Buisseret et al. (2020)</td><td>CNN -based AI (only use)</td><td>• AUROC: NR
• Sen: 75%
• Spe: 75%</td><td>NR</td></tr>
<tr><td>Suzuki et al. (2020)</td><td>CNN (only use)</td><td>• AUROC: NR
• Sen: NR
• Spe: NR
• Acc: 64.7±2.3%</td><td>Best combination
• MMSE
• KES
• FIM</td></tr>
<tr><td>Gietzelt et al. (2014)</td><td>Decision Tree (only use)</td><td>• AUROC: 0.80
• Sen: 78.2%
• Spe: 71.2%</td><td>NR</td></tr>
</table>

Abbreviations: ACC: Accuracy; ADL: Activities of Daily Living; Al: Artificial Intelligence; BML Body Mass Index; CART: Classification and Regression Tree; CNN: Convolutional Neural Network; FIM: Functional Independence Measure; GBM: Gradient Boosting Machine; IADL: Instrumental Activities of Daily Living; KES: Knee Extension Strength; LR: Logistci Regression; MMSE:Mini-Mental State Examination; NR: Not Reported; RF- Random Forest; ROC: Area Under the Receiver Operating Characteristic Curve; Sen: Sensitivity; Spe: Specificity; STRATIFY: St. Thomas's Risk Assessment Tool In Falling Elderly Inpatients; XGBoost- Extreme Gradient Boosting
